# Urolithin A regulates gut: liver axis to ameliorate alcohol-associated liver disease

**DOI:** 10.3389/fphar.2025.1706111

**Published:** 2026-01-19

**Authors:** Sweta Ghosh, Rajbir Singh, Zachary Matthew Vanwinkle, Craig James McClain, Vatsalya Vatsalya, Bodduluri Haribabu, Praveen Kumar Vemula, Venkatakrishna Rao Jala

**Affiliations:** 1 Department of Microbiology and Immunology, Brown Cancer Center, Center for Microbiomics, Inflammation and Pathogenicity, University of Louisville, Louisville, KY, United States; 2 Department of Medicine, University of Louisville, Louisville, KY, United States; 3 Department of Pharmacology & Toxicology, University of Louisville, Louisville, KY, United States; 4 Hepatobiology & Toxicology Center, University of Louisville, Louisville, KY, United States; 5 Alcohol Research Center, University of Louisville, Louisville, KY, United States; 6 Robley Rex Louisville VAMC, Louisville, KY, United States; 7 Division of Gastroenterology, Hepatology and Nutrition, Department of Medicine, University of Louisville, Louisville, KY, United States; 8 Clinical Laboratory for the Intervention Development of AUD and Organ Severity, University of Louisville, Louisville, KY, United States; 9 NIAAA, National Institutes of Health, Bethesda, MD, United States; 10 Institute for Stem Cell Biology and Regenerative Medicine (inStem), GKVK campus, Bangalore, Karnataka, India

**Keywords:** alcohol-associated liver disease, aryl hydrocarbon receptor, gut barrier function, inflammation, steatosis, urolithin A

## Abstract

**Background and Aims:**

Excessive alcohol consumption poses a significant global health concern, ranking as the world’s third-largest risk factor for diseases and disabilities, contributing to 5.9% of all deaths worldwide. Among various disorders linked to alcohol misuse, alcohol-associated liver disease (ALD) is the most prominent. ALD patients often exhibit increased intestinal permeability, systemic inflammation, gut dysbiosis, and hepatic steatosis. No FDA-approved therapies are available to treat ALD or to resolve the pathological domains of alcohol-induced gut barrier dysfunction, inflammation, and steatosis. The goal of this study is to investigate the potential therapeutic role of the microbial metabolite Urolithin A’ (UroA), in alleviating ALD.

**Methods:**

Caco-2 (monolayer colon epithelial) cells and AML12 (hepatocytes) cells were used to test the protective activities of UroA against EtOH-induced gut barrier dysfunction and lipid accumulation *in vitro.* Preclinical ALD mouse models were used to test the therapeutic potential of UroA against EtOH exposure. Additionally, we generated cell-specific deletion mice with aryl hydrocarbon receptor (AHR) deletion to define the role of intestinal epithelial cell AHR in UroA-mediated protective activities against ALD.

**Results:**

The results presented here demonstrate the efficacy of UroA as a potential therapeutic agent to protect against EtOH-induced disruption of tight junction proteins, inflammation, and lipogenesis both *in vitro* and *in vivo* models.

**Conclusion:**

Our findings suggest that the simultaneous targeting of gut barrier dysfunction, inflammation, and hepatic steatosis by treatment with UroA may offer new possibilities for combating ALD. Moreover, our results suggest that UroA-mediated protective activities against EtOH-induced gut barrier dysfunction and inflammation in ALD are dependent on intestinal epithelial cell-AHR.

## Graphical Abstract

**Figure:**
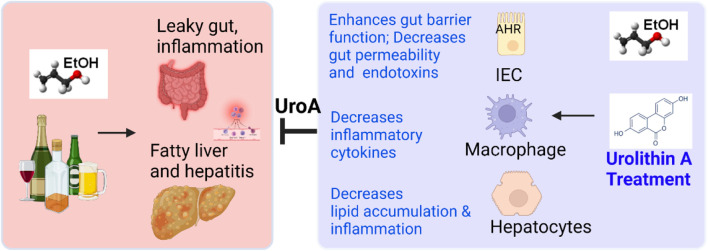
EtOH, Ethanol; AHR, Aryl hydrocarbon receptor; IEC, Intestinal epithelial cells.

## Highlights

- Patients with alcohol-associated liver disease (ALD) often exhibit increased intestinal permeability, gut dysbiosis, systemic inflammation, and hepatic steatosis.
- There are currently no available therapeutics that effectively target alcohol-induced gut barrier dysfunction and inflammation to mitigate ALD.
- The microbial metabolite, Urolithin A attenuates ALD in preclinical models by improving gut barrier function and decreasing hepatitis and steatosis.
- Urolithin A requires the intestinal epithelial cell-aryl hydrocarbon receptor to protect against EtOH-induced gut barrier dysfunction and inflammation in ALD.

## Introduction

Chronic and excessive alcohol intake is a leading driver of alcohol-associated liver disease (ALD), which remains a significant global health burden given its profound impact on liver-related morbidity and mortality (Leclercq et al., 2014). Approximately two million people die from liver disease each year, with nearly half of these deaths resulting from ALD (Engen et al., 2015; Bajaj, 2019). ALD comprises a spectrum of pathological conditions ranging from simple steatosis to more severe forms of liver injury, including hepatitis, cirrhosis, and, in some cases, hepatocellular carcinoma (Konturek et al., 2018; Llopis et al., 2016; Quigley et al., 2013; Rao, 2009; Parlesak et al., 2000). Despite its global impact, the precise mechanisms driving ALD remain incompletely understood, and therapeutic options are extremely limited. While the liver is the principal organ affected by chronic alcohol intake, the gastrointestinal (GI) tract is also markedly impacted, owing to its role as the initial site of alcohol absorption and metabolism. Therefore, understanding and mitigating alcohol-induced gut barrier dysfunction in ALD is critical to ameliorate ALD pathogenesis.

The GI tract is lined with a single layer of intestinal epithelial cells (IECs), firmly held together by tight junctions and adherens junction proteins, forming a crucial intestinal barrier (Hartsock and Nelson, 2008; Vancamelbeke and Vermeire, 2017). This barrier prevents the uncontrolled passage of various stressor/toxic molecules present in the intestinal lumen into systemic circulation. However, the excessive alcohol consumption has been linked to an increase in intestinal permeability, often referred to as “leaky gut,” due, in part, to the disruption of these barrier proteins (Hansson, 2020; Stärkel et al., 2018; Maccioni et al., 2023). Consequently, luminal contents, particularly microbial components, endotoxins, and microbes, pass through this compromised gut barrier and are subsequently translocated to the liver, resulting in inflammation and injury. This translocation triggers the activation of the innate immune system, leading to an increase in the production of proinflammatory cytokines and lipogenesis. Thus, the gut-liver axis contributes to the development and progression of ALD (Maccioni et al., 2020; Louvet and Mathurin, 2015).

Aryl hydrocarbon receptor (AHR) is a xenobiotic-sensing nuclear transcription factor that plays an important role in gut barrier integrity and intestinal inflammation (Stockinger et al., 2021; Kou and Dai, 2021). Recent studies have highlighted the importance of AHR signalling in the progression of ALD in preclinical models (Qian et al., 2022; Mackowiak and Gao, 2022). It was shown that exposure of EtOH led to decreased expression of intestinal AHR in mice and humans (Qian et al., 2022). In addition, *Ahr* deficiency in intestinal epithelial cells (IEC) aggravates EtOH-induced liver injury (Qian et al., 2022). Certain AHR activators, especially nutrient- and microbiota-derived AHR ligands have been shown to protect against EtOH-induced gut barrier dysfunction (Stockinger et al., 2021; Yu et al., 2018; Scott et al., 2020). Furthermore, interventions aimed at enhancing gut barrier integrity have been shown to ameliorate liver-related injuries and diseases in animal models (Plaza-Díaz et al., 2020; Chopyk and Grakoui, 2020). Evidence to alleviate the pathological AHR response, thereby treating ALD and resolving its pathological mechanisms is lacking.

Urolithins are microbial metabolites that are produced upon consumption of diets rich in ellagic acid (EA) or ellagitannins (ET), and are found in foods such as pomegranates, berries, and walnuts (García-Villalba et al., 2022; Tow et al., 2022; D’Amico et al., 2021; Ghosh et al., 2021). Among these urolithins, Urolithin A (UroA) has been extensively studied for its health-promoting effects (García-Villalba et al., 2022; D'Amico et al., 2021). Previously, we reported that UroA activates the AHR pathway and induces the expression of tight junction proteins in an AHR-NRF2 dependent manner, eventually protecting mice from colitis, inflammation, and gut barrier dysfunction (Singh et al., 2019; Ghosh et al., 2022a; Ghosh et al., 2022b; Ghosh et al., 2024a; Ghosh et al., 2024b). Furthermore, UroA treatment reduced triglyceride (TG) accumulation in adipocytes and hepatocytes (Kang et al., 2016). Recent studies also suggest that UroA can alleviate chronic alcohol-related liver disorders through a major urinary protein, 1 (MUP1) (Zhang et al., 2024), and alcohol-associated pancreatitis by inhibiting PI3K/AKT/mTOR signalling (Mehra et al., 2022). Although UroA, a well-known AHR modulator, has shown beneficial effects against metabolic disorders and gut inflammation, its efficacy in ALD and its role in AHR modulation in specific cell types have not been investigated in the context of ALD.

Here, we hypothesized that UroA protects against alcohol-mediated gut barrier dysfunction, and that treatment with UroA attenuates alcohol-related intestinal and liver damage. In this study, we used *in vitro* and *in vivo* models to investigate the cell-specific requirements of AHR in IECs and hepatocytes for UroA-mediated protection against ALD. Overall, our data demonstrated that UroA treatment protected against EtOH-induced intestinal barrier damage and hepatic steatosis in an intestinal epithelial AHR-dependent manner.

## Materials and methods

### Cell culture

Caco2 and AML12 cells (ATCC) were cultured in Eagle’s minimum essential medium (EMEM)-high glucose and Dulbecco’s Modified Eagle’s medium (DMEM)/F12 medium (Sigma Aldrich), respectively, supplemented with 10% fetal bovine serum, 1X penicillin-streptomycin solution (100X; P4333, Sigma Aldrich), and cultured at 37 °C in 5% CO_2_, 95% air in humidified incubator. AML12 cell culture medium was supplemented with ITS (insulin, transferrin, selenium; 1X, Sigma Aldrich) and dexamethasone (40 ng/mL; Sigma Aldrich). The incubators were saturated with alcohol during treatment to prevent evaporation. Breifly, closed container was used inside incubator with open dish of ethanol solution with twice the concentration in PBS that was used in cell culture medium of an experiment as described by Elamin et al. (2012).

### *In vitro* permeability assay

The Caco2 cells were cultured and maintained in EMEM-high glucose medium with 10% fetal bovine serum, 1X penicillin-streptomycin solution (100X; P4333, Sigma Aldrich) at 37 °C in 5% CO_2_, 95% air in humidified incubator. The *in vitro* permeability assay was performed as described by Singh *et al.* with slight modifications (Singh et al., 2019). Briefly, the cells were plated in 0.45 μm polyester membrane 24-well Transwell plates (Cat # 3470, Corning, United States) and grown for 21 days. The culture medium was changed every other day. The generated monolayer was treated with UroA (50 µM) for 1 h before the addition of insults, and then incubated for 24 h. After 24 h, the monolayers were washed with phosphate-buffered saline (PBS). Next, fluorescein isothiocyanate (FITC) -dextran (FD-4; Sigma) solution (200 µL of 1 mg/mL) in Hanks’ balanced salt solution (HBSS) was added to the apical chamber. Samples from the basal chamber were collected after 2 h, and the amount of FITC-dextran transported from the apical chamber to the basal chamber was calculated from the standard curve of FITC-dextran in HBSS, using a fluorescence plate reader (Ex. 480 nm Em. 525 nm). The insults used for barrier damage were lipopolysaccharide (LPS, O55:B5 Sigma; 50 ng/mL), TNF-α, IFN-γ (10 ng/mL each; Peprotech), EtOH (40 mM; Sigma), and high-mobility group box-1 (HMGB-1, 500 ng/mL).

### Immunofluorescence

Caco2 cells were grown on 8-well chambered slides (Thermo Fisher Scientific). The cells were treated with UroA (50 µM) in the presence or absence of EtOH (40 mM) for 24 h. After incubation, cells were fixed with methanol for 10 min at 4 °C, followed by blocking in 3% BSA and overnight incubation with primary antibodies against the tight junction proteins, zonula occludens (ZO-1) (1:100) and occludin (OCLN). The next day, the cells were washed with PBST and incubated with fluorescently labelled (Alexa Fluor 594 and Alexa Fluor 488) secondary antibodies (1:500 dilution; Thermo Fisher Scientific) for 2 h. The cells were then washed with PBS, and the nuclei were stained with DAPI (Sigma Aldrich). Confocal images were captured using a Nikon A1R confocal microscope with the appropriate laser channels.

### Staining of lipid droplets

Lipid accumulation in AML12 cells was determined by Oil Red O Staining and BODIPY staining. Cells were plated on 8-well chambered slides (Thermo Fisher Scientific) and cultured as described above. cells were treated with EtOH (40 mM) in the presence or absence of UroA (50 µM) for 24–48 h at 70% confluency. After incubation, cells were fixed with 4% paraformaldehyde at 4 °C for 30 min. After treatment, cells were washed with PBS, followed by the addition of Oil-Red -O solution (0.05%) and incubation for 15 min cells were then rinsed with 60% isopropanol (20 s) followed by rinsed with distilled water, and images were taken using an epifluorescence microscope (Keyence Digital Microscope). For BODIPY staining, cells were fixed with 4% paraformaldehyde and stained with 1 µM of BODIPY Green for 10 min at room temperature in the dark. After washing with PBS and coverslip mounting, the images were captured using a Nikon A1R confocal microscope.

### Real-time PCR

RNA was extracted using the Maxwell® 16 LEV simplyRNA cell/tissue kit (Promega), and cDNA was transcribed using 1 µg RNA using the TaqMan Reverse Transcription Kit (Applied Biosystems, CA, United States). After dilution, cDNA was used for real-time PCR by mixing with 100 nM gene-specific primers and 1X SYBR green Reaction Mix (Power SYBR® Green PCR Master Mix; Applied Biosystems) using a CFX96^TM^ Real-Time System (Bio-Rad). Relative changes in gene expression (fold-change) were calculated using the 2^−ΔΔ^CT method after normalization with the control (vehicle-treated).

### Western blots

After harvesting, cells or tissues were lysed in RIPA buffer (Sigma-Aldrich) containing protease inhibitors, and total protein lysates were collected as described previously (Ghosh et al., 2022c). The total protein was quantified using a BCA Protein Estimation Kit (Thermo Scientific). The lysates were loaded onto NuPAGE^TM^ 4%–12% Bis-Tris gel (Novex Life Technologies), transferred onto a polyvinylidene difluoride membrane (0.22 μm pore; Millipore), and blocked with 5% (w/v) skim milk powder (in Tris-Buffered Saline with Tween 20 (TBST) buffer). The Membranes were incubated with primary antibodies (4 °C overnight). The next day, the membrane was washed with TBST and incubated with the respective horseradish peroxidase-conjugated secondary antibodies. Protein bands were detected using a chemiluminescent substrate on a gel documentation system (ImageQuant LAS 4000). The blots were quantified using the ImageJ software.

### Mice

C57BL/6 mice were bred in the animal facility at the University of Louisville, Louisville, KY, United States. *Ahr* ^*fl/fl*^ (stock no. 006203) and *Villin-Cre* (stock no. 004586) on C57BL/6 background were purchased from Jackson Laboratories. For IEC-specific deletion of *Ahr, Ahr* ^*fl/fl*^ mice were crossed with *Villin-Cre* mice to obtain *Ahr* △ ^IEC^ mice. To validate the *Ahr* △ ^IEC^ mice, We used qPCR, immunoblotting, and immunofluorescence to validate the *Ahr* ^△ IEC^ mice. Animals were kept in temperature- and humidity-controlled rooms with a 12-h alternate light and dark cycle under specific pathogen-free barrier conditions. The animals had access to food and water *ad libitum*. Animals were acclimated to the experimental room for at least 4 days before the start of the study. The animals between ages of 8–10 weeks were used for all experiments, with protocols approved by the Institutional Animal Care and Use Committee (IACUC) of the University of Louisville. Experimental mice were treated with UroA or Vehicle (1% Carboxymethyl Cellulose (CMC), 0.1% Tween-80) with presence or absence of alcohol feeding. Control mice were treated with vehicle, but were not exposed to EtOH.

### Acute model of alcohol-associated liver disease

Male and female mice (8–10 weeks) were exposed to three consecutive doses of binge alcohol (5 g/kg) every 12 h. The mice were treated orally with UroA (20 mg/kg) or vehicle (1% CMC and 0.1% Tween-80) after every alcohol dose. Animals were euthanized by CO_2_ asphyxiation 4 h after the final alcohol dose. Blood was collected from the abdominal aorta, and serum was obtained, flash-frozen in liquid nitrogen, and stored at −80C until analysis. Tissues (the small intestine, colon, and liver) were collected for further analysis.

### Chronic model of alcohol-associated liver disease

Mice (8–10 weeks) were first fed a control liquid Lieber-DeCarli diet for 5 days to acclimatize the animals to a liquid diet. After 5 days, animals were ramped from 0%–5% alcohol-containing liquid diet over 1 week, and the 5% alcohol diet was continued for the next 4 weeks. Control animals were pair-fed isocaloric maltose dextrin. UroA (20 mg/kg, q.d.) was orally administered every other day during the alcohol treatment. At the end of the experiment, mice were euthanized by CO_2_ asphyxiation. Blood was collected from the abdominal aorta, and serum was obtained, flash frozen in liquid nitrogen, and stored at −80 °C until analysis. Other tissues (the small intestine, colon, and liver) were collected and stored for further analysis.

### Chronic plus binge model

In this model, 10–12-week-old *Ahr* ^*fl/fl*^ and *Ahr* △ ^*IEC*^ mice (both male and female mice) were first acclimatized to a liquid diet by feeding them a Lieber-DeCarli control diet (Cat #F1259)for 5 days as described previously (Bertola et al., 2013). The control diet was followed by Lieber-DeCarli liquid ethanol diet (Cat# F1258) supplemented with 5% ethanol for 10 days. Control animals were pair-fed an isocaloric liquid diet supplemented with maltose dextrin. On day 16, a single binge of EtOH (5 g/kg) was orally administered to the ethanol fed group, and maltodextrin solution (45% w/v) was provided to pair-fed group animals. For UroA treatment, the animals were orally gavaged with UroA (20 mg/kg, q.d.) on alternate days, starting from the initiation of the ethanol-fed diet. Animals were euthanized by CO_2_ asphyxiation 9 h after the final EtOH dose. Serum and organs of interest (small intestine, colon, and liver) were harvested and stored (as described above) for further analysis.

### *In vivo* intestinal permeability

Animals were orally administered a FITC-dextran (4 kDa) solution (60 mg/100 g body weight) 4 h before killing. Blood was collected and serum was obtained after centrifugation of clotted blood at 3500 × g for 15 min at 4 °C. Serum levels of FITC-dextran were calculated by measuring fluorescence in serum (Exc. 485 nm, Emm. 525 nm) and plotting the value against a standard curve of FITC-dextran generated in the mouse serum.

### Analysis of proinflammatory cytokines (IL-6, TNFα and IL-1β), ALT and AST

Serum cytokine levels (IL-6, TNF-α, and IL-1β) were measured using ELISA kits (BioLegend), according to the manufacturer’s instructions. Similarly, serum ALT and AST levels were measured using ALT/AST kits (Sigma), following the manufacturer’s instructions provided with the kits. To measure hepatic inflammatory cytokines, the liver was weighed, homogenized in 1X PBS, and centrifuged (12,000 × g for 15 min at 4 °C), the levels of cytokines (IL-6, TNF-α, and IL-1β) in the supernatant were measured using respective ELISA assay kits. Briefly, the liver was weighed and homogenized in solutions provided with the respective kits and the samples were centrifuged (12,000 × g for 15 min at 4 °C). The ALT and AST levels were determined.

### Endotoxin and fecal albumin level

To assess the effect of UroA on intestinal permeability, serum endotoxin and fecal albumin levels were determined. Serum endotoxin levels were used to provide mucosal to serosal permeability, whereas fecal albumin provided serosal to mucosal permeability. Serum endotoxin levels were determined using a chromogenic Limulus Amebocyte Lysate (LAL) assay kit (Thermo Fisher; Cat # 88282) following the manufacturer’s instructions. For fecal albumin level, feces were homogenized in homogenization buffer (50 mM Tris, 0.14M NaCl, 0,05% Tween 20, pH 8.0) at 100 mg/mL (w/v) and centrifuged at 1000 × g for 5 min to pellet down any debris and insoluble material. Albumin levels were quantified using a mouse albumin ELISA assay kit (Bethyl Labs, Cat # NC0096469) according to the manufacturer’s instructions.

### Liver triglyceride (TG) analysis

To the pre-weighed liver (100–300 mg), ethanolic KOH (2 parts ethanol: 1 part 30% KOH) was added (350 µL) and the sample was incubated at 55 °C overnight. The sample was vortexed and a water: ethanol (1:1) mixture was added to 1 mL. The sample was centrifuged at 8000 *g* for 10 min. The supernatant was collected, and a final volume (1200 µL) of a water:ethanol mixture was made up; 200 µL was collected from this sample, and 215 µL of 1 M MgCl_2_ was added to the sample. After incubation for 10 min on ice, samples were centrifuged at 8000 *g* for 10 min, 30 µL of supernatant was added to triglyceride reagent A (1 mL; Sigma), incubated for 15 min, and absorbance was measured at 540 nm. For quantification, glycerol standards (Sigma-Aldrich) are expressed as triolein equivalents. Liver TG levels were calculated using the following formula: TG (mg/g tissue) = cuvette triolein-equivalent glycerol concentration (mg/dL) × (10/30) × (415/200)×0.012 (dL)/tissue weight (g) (Park et al., 2023).

### Histology and immunohistochemistry

Liver and intestinal samples were fixed in formalin and embedded in paraffin. Paraffin-embedded blocks were sectioned to prepare 5 μm slides and stained with Hematoxylin and Eosin (H&E). For histopathological analysis, the samples were submitted to a veterinary histopathologist who was blinded to the treatment groups. For immunohistochemistry, formalin-fixed paraffin-embedded intestinal tissues were sectioned and stained with an anti-ZO-1 or anti-CLDN1 antibody followed by Alexa Fluor 488 secondary antibody. Nuclei were stained with DAPI. Fluorescence images were captured using a Nikon A1R confocal microscope with the appropriate filters.

### Statistical analysis

Statistical analyses were performed using GraphPad Prism (version 10). All data are expressed as the mean ± SEM. Statistical significance was analyzed using unpaired Student’s t-test or one-way ANOVA, followed by either tukey’s *post hoc* test or Dunnett’s *post hoc* multiple comparisons test. Normal (Gaussian) distribution assumption was made. In case of ordinary two-way ANOVA, Šídák’s multiple comparisons test was performed. Effect of Ethanol and UroA were included in the two-way ANOVA.

## Results

### Urolithin A protects from alcohol-induced disruption of gut barrier function

**FIGURE 1:**
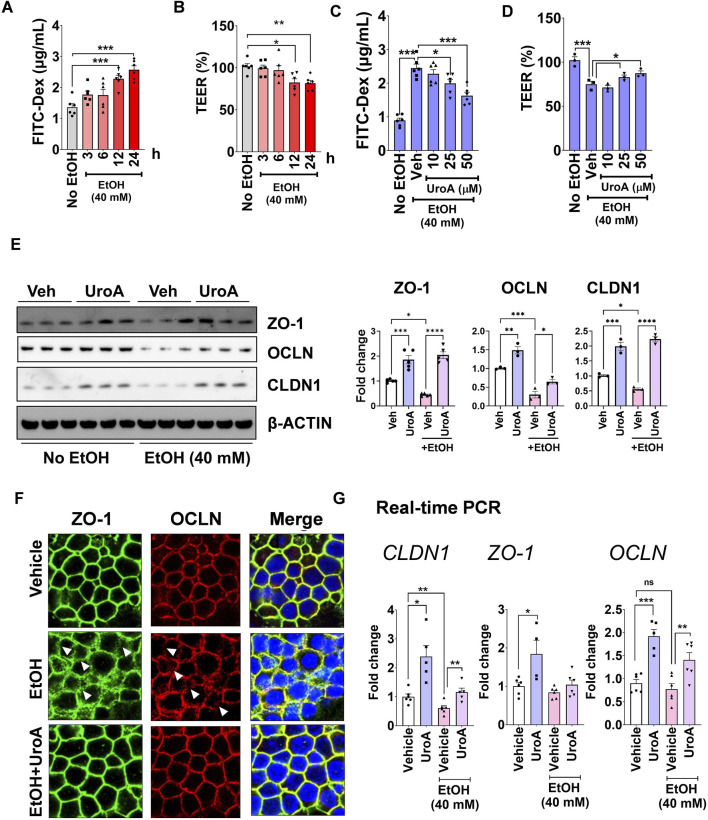
UroA protects against alcohol-induced gut barrier dysfunction. **(A,B)** Monolayered Caco2 cells on transwell membrane were exposed to EtOH (40 mM), and FITC-dextran permeability **(A)** and TEER values **(B)** were determined in time-dependent manner. **(C,D)**. Monolayered Caco2 cells on transwell membrane were pre-treated with vehicle or UroA (10, 25, 50 μM) for 24 h followed by the addition of EtOH (40 mM) for 24 h. FITC-dextran permeability assay **(C)** and the TEER values were determined **(D)**. **(E–G)** UroA protects against EtOH-induced tight junction (TJ) protein dysregulation. **(E)** Caco-2 cells were pre-treated with UroA (50 μM) 2 h prior to EtOH (40 mM) exposure and continued for additional 24 h. Expression of TJ proteins was analyzed. **(E)** Representative Western blots for ZO-1, OCLN, CLDN1 are shown. Western blots were quantified by ImageJ software. **(F)** Immunofluorescence confocal images of ZO1 and OCLN were captured using Nikon A1R confocal microscope. Red arrows indicate disrupted TJP. **(G)** Caco-2 cells were pre-treated with UroA (50 μM) 2 h prior to EtOH (40 mM) exposure and continued for 24 h mRNA levels of *OCLN*, *CLDN1* and *Z O -1* were quantified by real-time PCR using standard SyBR green method. One-way ANOVA test was performed using GraphPad Prism software. Error bars, ±SEM; ***p < 0.001; **p < 0.01.

ALD patients exhibit increased gut permeability leading to increased endotoxemia and systemic inflammation (Engen et al., 2015; Bajaj, 2019). To determine whether urolithins can prevent alcohol-induced gut permeability, we used Caco-2 colon epithelial cells grown on Transwell membranes. As expected, exposure to 40 mM EtOH for 24 h caused a significant increase in permeability, as shown by increased FITC-dextran permeability (Figure 1A) and decreased trans-epithelial electrical resistance (TEER) (Figure 1B). The gut microbiota metabolizes ellagic acid (EA) into urolithin (Uro) A, B, and C by sequential removal of hydroxyl groups (Tomas-Barberan et al., 2017) (Supplementary Figure S1A). To determine which urolithins are effective against EtOH-induced gut barrier damage, we treated colon epithelial cells (Caco-2) cultured as monolayers on transwell membranes with EA, UroA, UroB, or UroC. As shown in Supplementary Figures S1B,S1C, UroA treatment significantly reduced EtOH-induced epithelial permeability and restored TEER compared to EA, UroB, or UroC. Further studies are focused protective impact of UroA against alcohol-induced epithelial cell damage. As shown in Figures 1C,D, UroA showed a dose-dependent protective effect against EtOH-induced increased permeability and reduced TEER values. To further confirm this effect, we evaluated the effect of UroA on the expression of tight junction proteins that regulate epithelial barrier function (Horowitz et al., 2023; Groschwitz and Hogan, 2009). As shown in Figure 1E and Supplementary Figure S1D, ethanol exposure significantly decreased the expression of tight junction proteins (CLDN1, OCLN, and ZO-1), and treatment with UroA protected against alcohol-induced downregulation of tight junction proteins (TJP). Furthermore, in immunofluorescence staining of *Z O -1* and *OCLN* we observed that these tight junction proteins get internalized (marked with red arrowhead), rather than expression inhibition with alcohol treatment and UroA showed protective activities against it (Figure 1F). Moreover, mRNA levels of *Z O -1, OCLN* and *CLDN1* (Figure 1G), showed the protective effects of UroA.

### Treatment with UroA reduced EtOH-induced intestinal permeability in the binge model

**FIGURE 2:**
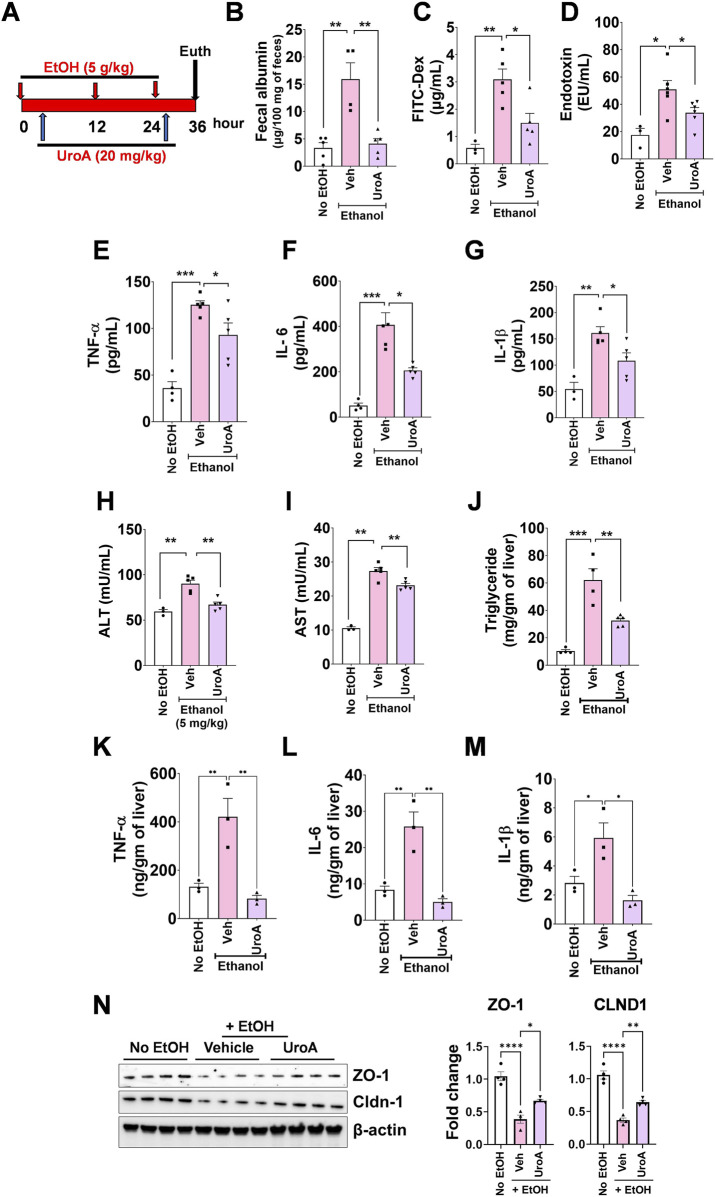
UroA protects against acute alcohol-induced gut barrier dysfunction and liver damage. **(A)** Female C57BL/6J (n = 5, 8–10 weeks old) mice were orally treated with EtOH (5 g/kg) at 0, 12 and 24 h. Mice were treated with Veh or UroA at 4 and 26 h and euthanized at 36 h. FITC-dextran was orally administered 4 h prior to euthanasia. **(B)** Fecal albumin. **(C)** Serum FITC-dextran. **(D)** Serum endotoxin. **(E)** Serum TNF-α. **(F)** Serum IL-6. **(G)** Serum IL-1β. **(H)** Serum ALT. **(I)** Serum AST. **(J)** Total triglycerides. **(K–M)** Liver inflammatory cytokines (IL-6, TNF-α, and IL-1β) were measured using appropriate ELISA kits. **(N)** Expression of tight junction proteins, ZO-1 and CLDN1 was measured by Western blots and quantified using Image J software.

Increased intestinal permeability and endotoxemia are important features of the pathogenesis of ALD. The therapeutic and preventive efficacy of UroA was tested in a murine model of acute binge ALD, as described previously (Bertola et al., 2013; Ghosh et al., 2018) (Figure 2A). Intestinal permeability was assessed using fecal albumin and *in vivo* fluorescein isothiocyanate (FITC)-dextran (4 KDa) permeability assays. As shown Figures 2B,C, EtOH induced increased fecal albumin excretion and FITC-dextran uptake into the blood. Oral treatment with UroA significantly protected against EtOH-induced increase in fecal albumin excretion and permeability to FITC-dextran. Furthermore, UroA treatment attenuated EtOH-induced increases in endotoxins (Figure 2D) and inflammatory cytokines (IL-6, TNF-α, and IL-1β) (Figures 2E–G). UroA treatment also reduced ethanol-induced elevations in ALT and AST levels in the binge model (Figures 2H,I). Ethanol gavage induced elevated TG levels in liver tissues, and treatment with UroA significantly reduced EtOH-induced triglyceride (TGs) levels in liver tissues (Figure 2J) and inflammatory cytokines (IL-6, TNF-α, and IL-1β) (Figures 2K–M). Importantly, EtOH exposure led to decreased TJPs such as ZO-1, CLDN1 and Occludin (OCLN) (Figure 2N; Supplementary Figure S2) and UroA treatment protected EtOH-induced tight junction proteins downregulation (Figure 2N; Supplementary Figure S2). These results suggested that UroA treatment protects against EtOH-induced gut barrier permeability, inflammation, and liver damage. We also tested whether pre-treatment with UroA has the similar protective activities against ALD using binge model. Our data indicated that UroA has similar protective activities EtOH-mediated gut permeability, inflammation and barrier dysfunction (Supplementary Figure S3).

### UroA treatment attenuated gut:liver dysfunction/injury in a model of chronic alcohol consumption

**FIGURE 3:**
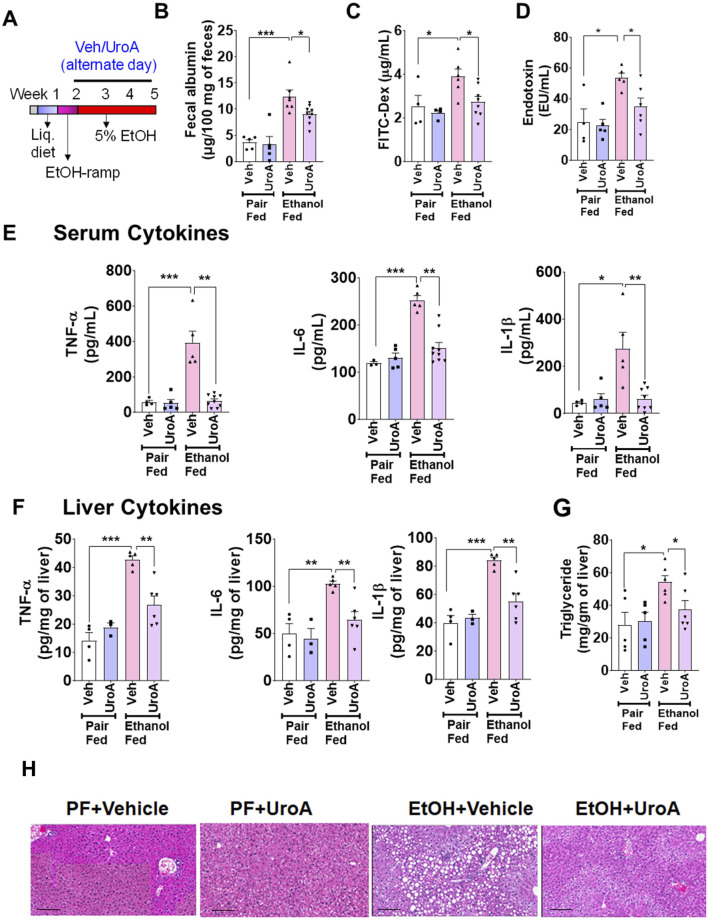
UroA protects against chronic alcohol-induced gut barrier dysfunction and liver damage. **(A)** C57BL/6 mice (8–10 weeks old) were used in chronic ALD models and analyzed after 4 weeks as described. Pair-fed (PF, n = 5/group) and EtOH-fed (n = 10/group) mice were treated with Veh (1%CMC+0.1% Tween-80) and UroA (20 mg/kg, q.d.) on alternate days for 4 weeks. **(B)** Fecal albumin was measured using an ELISA kit. **(C)** *In vivo* permeability was measured by determining serum levels of FITC-Dextran 4 h post oral gavage of FITC-dextran. **(D,E)** Indicated serum levels of endotoxins and cytokines were measured in serum using standard kits. **(F)** The levels of TNF-α, IL-6, and IL-1β in the liver were measured using ELISA methods. Liver total triglycerides **(G)** were measured using ELISAs. **(H)** H&E images of livers. Error bars, ±SEM. 2-Way ANOVA with multiple comparison testing was performed. *p < 0.05, **p < 0.01, ***p < 0.001.

**FIGURE 4:**
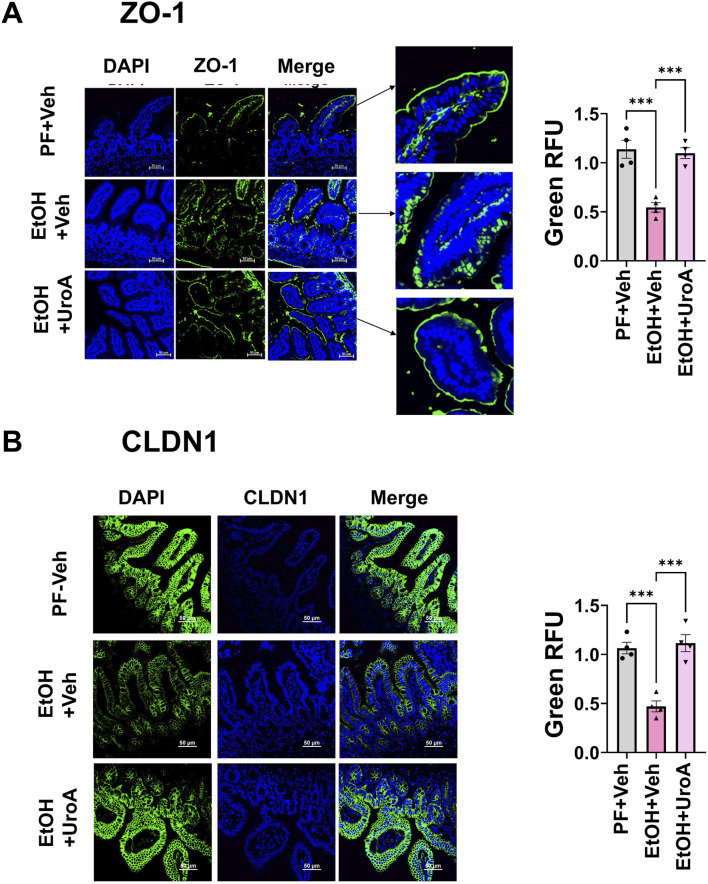
UroA treatment protects against alcohol-induced disruption of the tight junction protein, zonula occludens-1 (ZO-1) and CLDN1. 10-week-old C57BL/6 mice were used in chronic ALD models and samples were analyzed after 4 weeks as described. Pair-fed (PF, n = 5/group) and EtOH-fed (n = 10/group) mice were treated with Veh (0.25% CMC) and UroA (20 mg/kg) on alternate days for 4 weeks. The small intestines from these mice were processed and fixed. The paraffin sections were stained with ZO-1 **(A)** and CLDN1 **(B)** antibodies followed by appropriate secondary antibody tagged with Alexa-488 along with DAPI. The fluorescence images were captured using Nikon A1R confocal microscope using appropriate filters. Scale bar 50 μM. Fluorescence intensity was calculated by Image J. One-way ANOVA test was performed using GraphPad Prism software. Error bars, ±SEM; ***p < 0.001.

Next, we investigated whether UroA treatment protects against chronic ALD pathogenesis. (Figure 3A) (Singh et al., 2019). Chronic alcohol exposure significantly induced increased intestinal permeability, as evident from increased fecal albumin level, serum FITC-dextran and serum endotoxin levels compared to the vehicle-treated pair-fed group (Figures 3B–D), Treatment with UroA in ethanol-fed group reduced ethanol-induced gut permeability with reduced fecal albumin level, serum FITC-dextran and serum endotoxin levels compared to the vehicle-treated ethanol-fed group (Figures 3B–D). However, treatment with UroA in the pair-fed group did not alter the intestinal permeability. Increased blood endotoxin levels were associated with higher levels of proinflammatory cytokines such as IL-6, TNF-α and IL-1β. As shown in Figure 3E, EtOH-fed vehicle-treated group have displayed increased serum inflammatory cytokines compared to pair-fed mice. However, UroA treatment of EtOH fed mice exhibited reduced levels of these proinflammatory markers (Figure 3E) compared to those in the EtOH-fed vehicle-treated group. When alcohol is consumed for a longer period, it and its metabolites, along with microbial products from the intestine, are thought to affect the liver physiology and cause inflammation and fat deposition. Therefore, we analyzed whether UroA treatment protects against EtOH-induced liver inflammation and steatosis. Animals in the EtOH-fed group showed elevated levels of proinflammatory cytokines (IL-6, TNF-α, and IL-1β) and triglycerides in the liver compared to pair-fed animals. Furthermore, treatment with UroA significantly reduced EtOH-induced proinflammatory cytokine and triglyceride levels in the liver (Figures 3F,G). Histopathological analysis of the liver also revealed that UroA treatment significantly reduced hepatic steatosis in the EtOH-fed animals (Figure 3H). Further analysis of tight junction proteins by immunofluorescence staining revealed EtOH-induced disruption of tight junction proteins, and treatment with UroA protected the cells against EtOH-induced damage (Figure 4).

### UroA attenuates alcohol-induced steatosis in mice and AML12 cells administered alcohol

**FIGURE 5:**
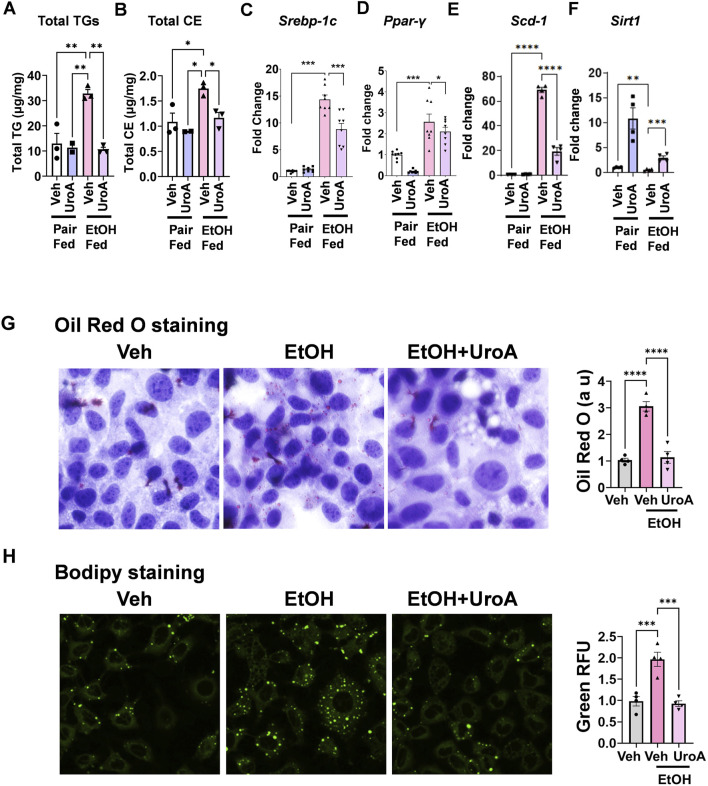
UroA treatment reduced hepatic steatosis. Total lipid panel (n = 3 per group) of livers of the mice fed with EtOH (5% EtOH in Lieber-DeCarli liquid diet and control diet (pair fed, PF) for 4 weeks) and were orally treated with UroA (20 mg/kg; q.d.) or vehicle (1%CMC+0.1% Tween-80) on alternate days. **(A)** Total levels of triglycerides (TG), **(B)** cholesterol ester (CE) were separated on TLC and measured using Agilent 7890A gas chromatography. **(C–F)** mRNA levels of *Srebp-1c, Ppar-γ, scd-1* and *sirt-1* were measured in the livers exposed to EtOH and treated with UroA or Vehicle as described above. **(G,H)** AML12 cell line was used as model system to examine the direct effects of UroA on lipid accumulation. AML12 cells were treated with EtOH (40 mM) in the presence or absence of UroA (50 µM). Lipids were stained using standard Oil Red O **(G)** or BODIPY **(H)** staining procedures. Error bars, ±SEM. 2-way ANOVA was performed with multiple comparison adjustments. *p < 0.05, **p < 0.01, ***p < 0.001.

Acute and chronic ethanol consumption results in the acceleration of fatty acid, triglyceride, and phospholipid synthesis in the liver (Lieber and Savolainen, 1984). We examined the total lipid content using lipidomic analysis. As shown in Figures 5A,B, the total triglyceride (TGs) and total cholesterol ester (CEs) levels were significantly increased in EtOH-fed mice and significantly decreased with UroA treatment. Sirtuin 1 (SIRT1), an NAD^+^-dependent deacetylase, inhibits hepatic lipogenesis, stimulates fatty acid β-oxidation, and maintains cholesterol and bile acid levels (Ren et al., 2020). It has been reported that both EtOH-mediated inhibition of SIRT1 and deletion of SIRT1 promote the development of alcohol-associated fatty liver (Ren et al., 2020; You et al., 2015; Yin et al., 2014). UroA treatment has been shown to activate the sirtuin 1 (Sirt1)-AMP-activated kinase (AMPK) pathway in a variety of cells and models, including BV2 microglia (Velagapudi et al., 2019), adipocytes, and hepatocytes (Kang et al., 2016). Therefore, we investigated whether UroA treatment downregulated EtOH-induced lipid accumulation in the liver by upregulating the sirtuin 1 (Sirt1)-AMP-activated protein kinase (AMPSIR1) pathway. SIRT1-AMPK disrupts signaling pathways mediated largely by various transcriptional regulators and several co-regulators, including SREBP-1c and PPARγ (Ren et al., 2020; You and Arteel, 2019). Expression analysis of *Srebp-1*, *Ppar-γ*, and *Scd-1* in mouse livers showed that ethanol-fed mice had higher levels of these genes compared with pair-fed controls. UroA treatment reduced the ethanol-induced upregulation of *Scd-1*, *Srebp-1*, and *Ppar-γ* in the liver (Figures 5C–E). Ethanol exposure led to a reduction in *Sirt-1* expression, whereas UroA treatment effectively restored it (Figure 5F).

To validate and further investigate the effects of UroA on hepatic triglyceride (TG) accumulation observed in *in vivo* ALD models, we performed *in vitro* experiments using AML12 liver cell lines. AML12 cells were established from hepatocytes of CD1 mice transgenic for human TGF-α (MT42 line) and represent a physiologically relevant model for studying hepatocyte function. EtOH-induced lipid droplets were detected using Oil Red O and neutral lipid accumulation using BODIPY staining methods. As shown in Figures 5G,EtOH treatment increased Oil Red O staining (several small oil droplets) and BODIPY staining (punctate green stain) (Figure 5H) compared with vehicle treatment in AML12 cells. UroA treatment significantly reduced EtOH-induced lipid droplets in both assays. These results suggested that UroA acts directly on hepatocytes and plays an important role in reducing steatosis.

### UroA mediates gut protective activities in an AHR-dependent manner in an acute short-term model of alcohol-induced organ injury

**FIGURE 6:**
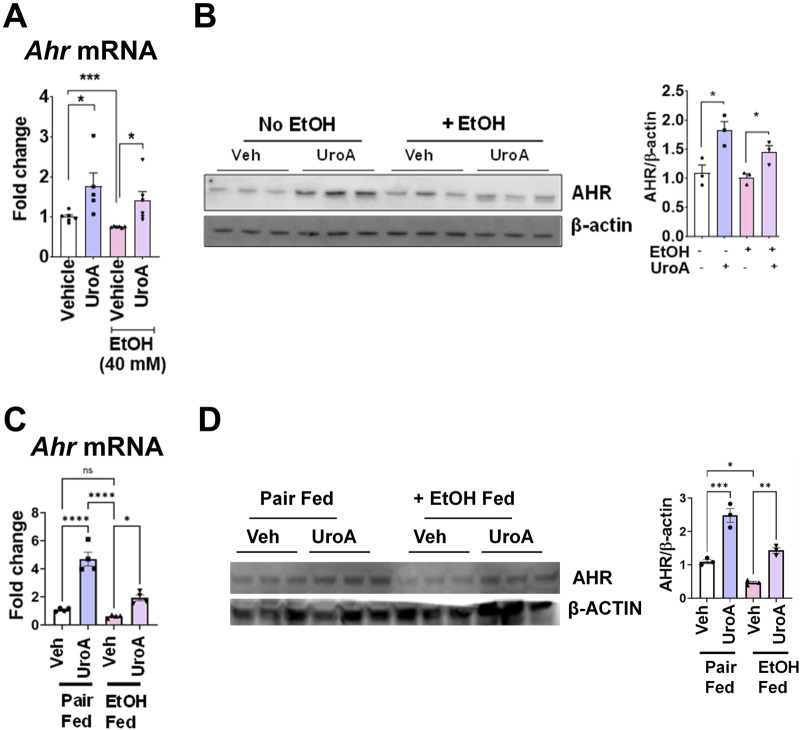
UroA rescues EtOH-induced downregulation of AHR in the intestines. **(A)** Monolayered Caco2 cells at 80% confluence were pre-treated with vehicle or UroA (50 μM) for 24 h followed by the addition of EtOH (40 mM) for 24 h. Total RNA was isolated, and levels of AHR mRNA were determined using real-time PCR utilizing the SyBR green method. **(B)** AHR expression at the protein level was evaluated by Western blot, and band intensities were quantified by ImageJ software. **(C,D)** C57BL/6J mice were fed with EtOH (5% EtOH in Lieber-DeCarli liquid diet for 5 weeks) or control diet (pair fed, PF) and orally treated with UroA (20 mg/kg) or vehicle (1%CMC+0.1% Tween-80) on alternate days. The intestines from these mice were dissected and prepared for tissue lysates. Expression of *Ahr* was evaluated by the SyBR green qPCR method **(C)** and AHR protein levels were evaluated by Western blot method **(D)**. Images were quantified by Image J. Error bars, ±SEM. 2-way ANOVA with multiple comparisons was performed. *p < 0.05, **p < 0.01, ***p < 0.001.

We and others have shown that UroA acts as an AHR ligand (Singh et al., 2019; Muku et al., 2018) and potentially mediates several functional activities in an AHR-dependent manner. In our previous study, we demonstrated that UroA induced tight junction proteins in an AHR-NRF2-dependent manner (Singh et al., 2019). To further examine the effects of UroA on the AHR pathway in the presence of alcohol, Caco-2 colon epithelial cells were used. As shown in Figure 6, EtOH treatment significantly reduced the expression of AHR at mRNA level (Figure 6A) and but not at protein levels (Figure 6B). Cells treated with UroA were significantly protected from the EtOH-induced downregulation of AHR. Furthermore, we evaluated AHR expression in the intestines of mice fed a chronic alcohol diet. As shown in Figure 6C, chronic consumption of EtOH led to the downregulation of AHR in mouse intestines, and UroA treatment restored or upregulated AHR expression in the intestines. EtOH has significantly reduced AHR expression at protein level in ethanol fed mice, whereas UroA upregulated the AHR expression (Figure 6D).

**FIGURE 7:**
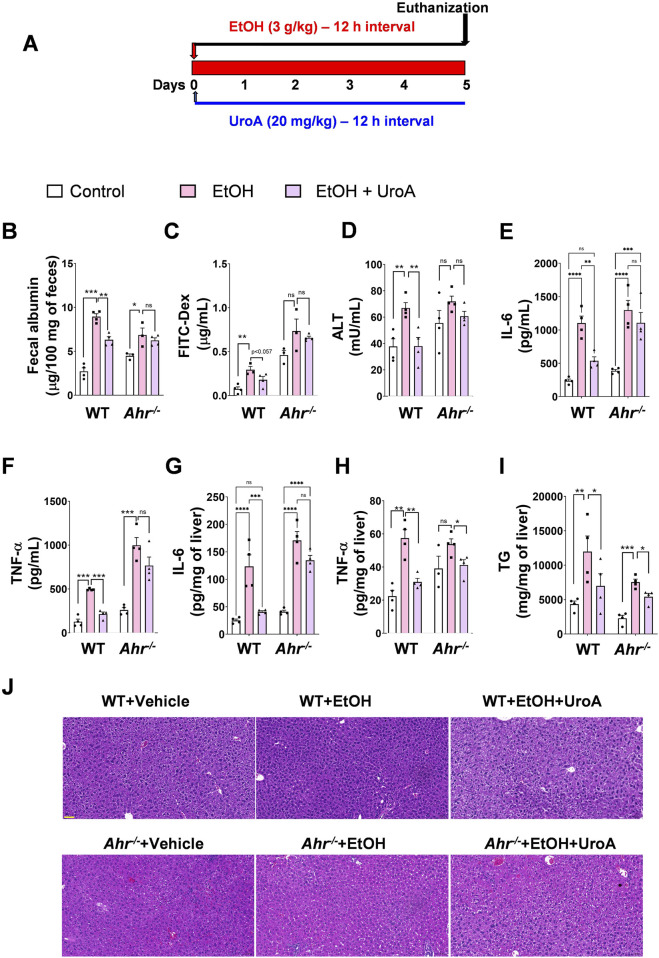
UroA mediates ALD protective activities in an AHR-dependent manner. **(A)** C57BL/6 and *Ahr* ^*−/−*^ mice were subjected to acute alcohol insult. The mice were treated with alcohol (3 g/kg) for 5 days at 12 h intervals through oral gavage. Mice were treated with UroA (20 mg/kg) at 12 h intervals until euthanasia. **(B)** Albumin levels in fecal samples were determined using ELISAs. **(C)** *In vivo* permeability was measured by determining serum levels of FITC-Dextran 4 h post oral gavage of FITC-dextran. Serum ALT **(D)**, IL-6 **(E)**, TNF-α **(F)**, levels were measured by standard ELISA methods. Liver IL-6 **(G)**, TNF-α **(H)** and triglycerides (TGs) **(I)** were measured by ELISAs using appropriate kits. **(J)** The representative H&E images of livers are shown. Error bars, ±SEM. 2-way ANOVA with multiple comparisons was performed. *p < 0.05, **p < 0.01, ***p < 0.001.

To determine the role of AHR in UroA-protective activities against ALD, we subjected *Ahr* ^*−/−*^ mice to acute alcohol insult. Since *Ahr* ^*−/−*^ mice display increased gut permeability and are more susceptible to inflammatory disorders, we used a lower dose of EtOH (3 g/kg) for 5 days at 12 h intervals through oral gavage to prevent mortality with the alcohol gavage. Mice were treated with UroA (20 mg/kg) at 12 h intervals as well (Figure 7A). As expected, EtOH exposure led to increased gut permeability, which was evident from the increased albumin levels (Figure 7B) and elevated FITC-dextran levels (Figure 7C) in EtOH-exposed animals. Treatment with UroA reduced EtOH-induced gut permeability in wild-type mice, but not in *Ahr* ^*−/−*^ mice (Figures 7B,C). Furthermore, UroA treatment reduced EtOH-induced serum ALT and TNF-α, IL-6 as well as liver TNF-α, IL-6 and TGs in wild-type mice, but not in *Ahr* ^*−/−*^ mice (Figures 7D–H). It is important to note that *Ahr* ^−/−^ mice have lower TG levels; however, the fold change is similar to that observed in WT animals. Interestingly, EtOH appeared to have a surprisingly limited impact on the livers of *Ahr* ^*−/−*^ mice, as assessed by ALT levels. Importantly, UroA treatment did not cause significant changes in *Ahr* ^*−/−*^ mice compared with wild-type mice. Triglycerides (TG) and histological analysis of the livers further indicated that low doses of EtOH did not cause a significant accumulation of lipids in the livers of either wild-type or *Ahr* ^*−/−*^ mice (Figures 7I,J).

### Intestinal AHR is required for UroA-mediated protective activities in the NIAAA chronic binge experimental model of ALD

**FIGURE 8:**
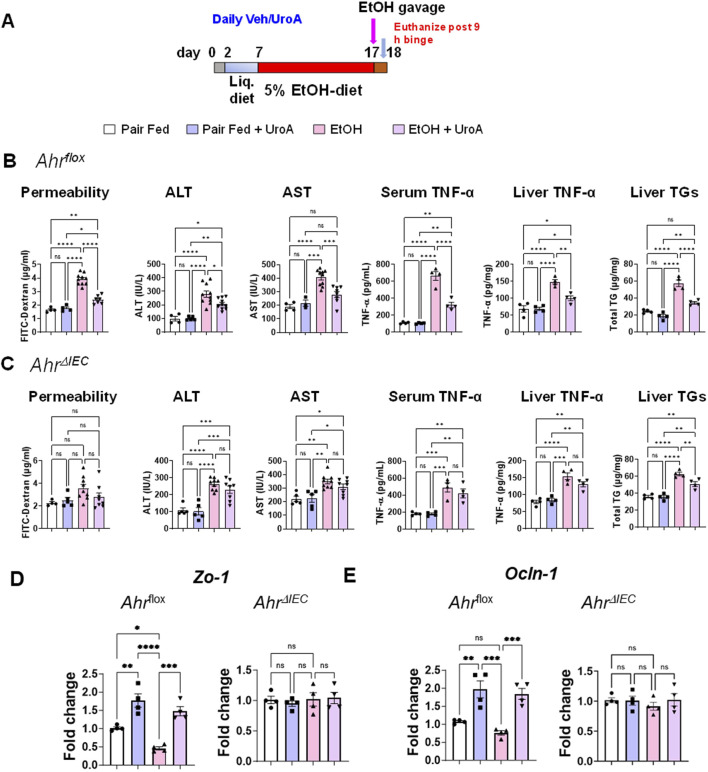
UroA mediates ALD protective activities in intestinal epithelial cells (IECs) in an AHR-dependent manner. **(A)** *Ahr* ^*flox*^ and *Ahr* ^*ΔIEC*^ (*Ahr* deleted in IEC) mice (n = 8–10 per group) were subjected to the 10 + 1 chronic + binge ALD model, and mice were treated with vehicle or UroA (20 mg/kg) every alternate day. **(B,C)** Intestinal permeability (FITC-Dextran assay), ALT, AST, serum and liver TNF-α and liver TGs were measured in *Ahr* ^*flox*^ and *Ahr* ^*ΔIEC*^ mice as described in methods. **(D,E)** UroA-mediated restoration of TJP is dependent on IEC-AHR. Total RNA from the colons of *Ahr* ^*flox*^ and *Ahr* ^*ΔIEC*^ mice that were subjected the 10 + 1 binge alcohol model was isolated. The mRNA levels of zonaoccludin-1 (*Zo-1)* **(D)** and occludin (*Ocln*) **(E)** were measured using the SyBR green method. Error bars, ±SEM. 2-way ANOVA with multiple comparisons was performed. *p < 0.05, **p < 0.01, ***p < 0.001.

ALD pathogenesis involves the gut-liver axis, including gut barrier dysfunction, hepatic steatosis, and inflammation with subsequent liver damage. As shown above, UroA protects against EtOH-induced gut barrier dysfunction and steatosis in an AHR-dependent manner. However, the cell-specific requirements of AHR for UroA-mediated protective activity are unknown. To evaluate the cell-specific requirement of AHR for UroA-mediated protective activities, we used *Ahr-villin* ^*Cre/+*^ mice (*Ahr* deleted in intestinal epithelial cells, *Ahr* ^*ΔIEC*^) (Supplementary Figure S4) along with control *Ahr* ^*flox*^ mice. *Ahr* ^*flox*^ and *Ahr* ^*ΔIEC*^ mice were subjected to a well-established 10 + 1 binge alcohol model as described previously. These mice were fed a Lieber-DeCarli liquid diet (5% EtOH) for 10 days, followed by a single oral gavage of ethanol (5 g/kg body weight, or maltose dextrin as control) on day 11, and mice were euthanized 9 h after binge (Figure 8A). *Ahr* ^*flox*^ mice displayed a phenotype similar to that of wild-type (C57BL/6) mice, in which EtOH exposure led to increased intestinal permeability, TNF-α, ALT, AST, and TGs levels. Moreover, treatment with UroA significantly downregulated EtOH-induced intestinal permeability, inflammation, and steatosis, indicating that UroA treatment attenuated ALD even in the 10 + 1 binge model (Figure 8B). However, UroA treatment failed to protect against EtOH-induced intestinal permeability, ALT, AST and inflammatory cytokines in *Ahr* ^*ΔIEC*^ mice, suggesting that UroA mediates its activities through intestinal AHR (Figure 8C). Interestingly, we observed, very low but significant reduction of TG in *Ahr* ^*ΔIEC*^ mice upon UroA treatment when exposed to EtOH. It is possible that UroA may direct impact on liver independent of IEC-AHR. Further studies are require to address this hypothesis. Next, we have evaluated the expression patterns of tight junction proteins (zonula occludens-1 (*Zo-1)* and occludin (*Ocln)-1*) in these mice. As shown in Figure 8D, EtOH-exposed Ahrflox mice displayed reduced expression patterns in *Ahr* ^*flox*^ mice, and UroA treatment protected against EtOH-induced reduction of tight junction proteins. Interestingly, we did not observe significant changes in the tight junction protein expression patterns in *Ahr* ^*ΔIEC*^ mice treated with EtOH or UroA.

**FIGURE 9:**
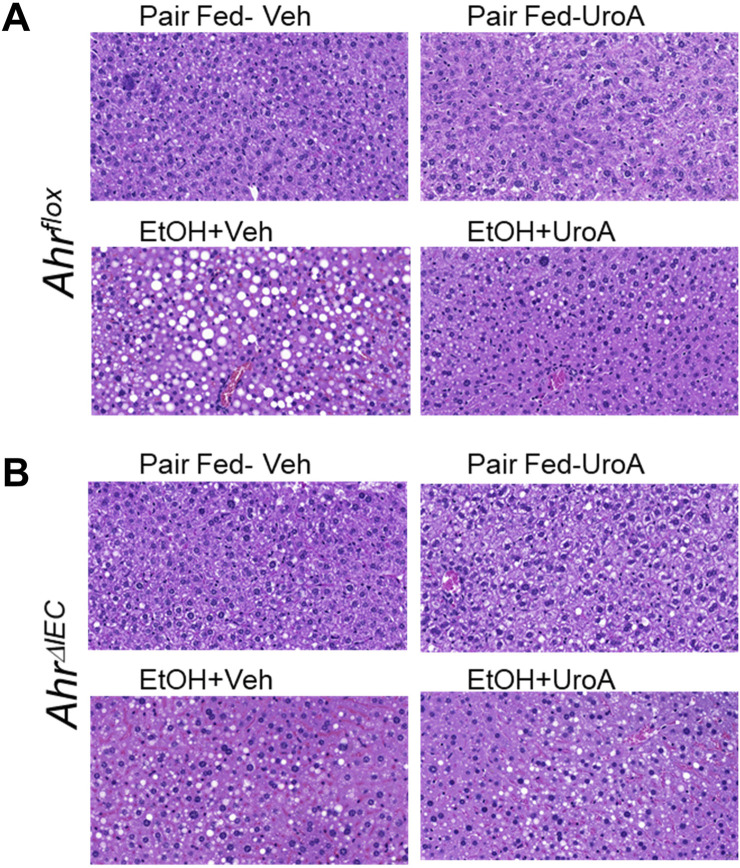
H&E analysis of livers from mice subjected to the 10 + 1 chronic + binge ALD model. Formalin fixed livers of mice (*Ahr* ^*flox*^ **(A)** and *Ahr* ^*ΔIEC*^ **(B)**) that were subjected to the 10 + 1 binge alcohol model were paraffin-embedded and H&E staining was performed. These mice were treated by oral gavage every alternate day with Vehicle or UroA (20 mg/kg; q.d.). Images were scanned using a PanDesk scanner and representative parts of the liver were captured.

H&E staining analysis of livers also suggested that UroA treatment reduced steatosis in *Ahr* ^*flox*^ mice, but not in *Ahr* ^*ΔIEC*^ mice (Figure 9). It is possible that the deletion of *Ahr* potentially resulted in decreased levels of harmful EtOH-induced metabolites in the liver, leading to reduced steatosis.

## Discussion

Excess alcohol consumption is the world’s third leading risk factor for diseases and disabilities, accounting for 5.9% of all deaths worldwide (Organization Wh, 2014). Despite its high prevalence and profound economic and health impacts, the clinical management of patients with ALD largely relies on lifestyle changes, including nutritional support and abstinence (Mackowiak et al., 2024). Current therapies targeting inflammation alone may not be sufficient to ameliorate the underlying cause of alcohol-induced tissue damage. Steroids, the only available treatment for ALD, can only be used for alcohol-associated hepatitis (with conditions when Maddrey is >32), are not targeted, and cannot be used for other forms and stages of ALD. Chronic alcohol consumption not only causes liver damage but also causes gut dysbiosis and increased intestinal permeability, leading to enhanced endotoxemia and inflammation (Engen et al., 2015; Bajaj, 2019). These key pathogenic events can lead to disease progression from steatosis to steatohepatitis, fibrosis, cirrhosis, and potentially hepatocellular carcinoma (Konturek et al., 2018; Llopis et al., 2016; Quigley et al., 2013; Rao, 2009; Parlesak et al., 2000; Groschwitz and Hogan, 2009; Rao et al., 2004; Zhou and Zhong, 2017; Wang et al., 2014; Donohue, 2007; Yang et al., 2019). Alcohol-induced intestinal permeability, chronic low-grade inflammation, and steatosis form a self-perpetuating loop that enhances inflammatory burden and compromises liver function. Thus far, there are no non-toxic and safe drugs available to target both gut barrier dysfunction and liver inflammation/injury. Therefore, understanding the pathogenesis and finding novel therapies to ameliorate ALD would have a significant impact on the overall health of this large patient population. We identified UroA as a potentially effective and safe medication for ALD.

Recent studies have highlighted that the overall benefits of pomegranate dietary supplements were due to further downstream metabolites, called “urolithins,” generated from gut microbiota (Tomas-Barberan et al., 2017; Cerda et al., 2004; Cerda et al., 2003; Doyle and Griffiths, 1980; Nunez-Sanchez et al., 2014; Larrosa et al., 2006). Urolithins have a much higher absorption rate than polyphenolics, ellagic acid, and ellagitannins because of their increased lipophilicity and improved bioavailability (Espin et al., 2007). Among urolithins, Urolithin A (3,8-dihydroxy-6H-benzo [c]chromen-6-one, UroA) displayed the most potent anti-inflammatory, antioxidant, and anti-aging properties compared to other metabolites (Espin et al., 2013; Tomas-Barberan et al., 2016; Ryu et al., 2016; Saha et al., 2016). Blood concentrations of UroA can reach micromolar levels without displaying toxicity (Tomas-Barberan et al., 2017; Espin et al., 2007; Larrosa et al., 2010; Gonzalez-Sarrias et al., 2010; Heilman et al., 2017). Recently, our group demonstrated that treatment with UroA enhances gut barrier function, in addition to blocking inflammation, protecting against colitis development (Singh et al., 2019), and blocking neutrophil recruitment and function (Saha et al., 2016). Independent studies suggested that only 40%–50% of human subjects can produce UroA despite consuming the same amounts of EA/ET-rich diets at different levels (Cerda et al., 2004; Nunez-Sanchez et al., 2014; Gonzalez-Sarrias et al., 2010; Cerda et al., 2005a; Garcia-Villalba et al., 2013; Tomas-Bar et al., 2014; Cerda et al., 2005b; Seeram et al., 2008; Seeram et al., 2006; Cortés-Martín et al., 2018; Gonzalez-Sarrias et al., 2017). These differences can be attributed to the presence or absence of the microbiota responsible for UroA production. Thus, the consumption of EA/ET-rich diets and the presence of specific microbes that convert them into beneficial metabolites are critical for their health effects. Reduced levels of beneficial microbial metabolites may compromise the gut barrier function, leading to endotoxemia and liver inflammation/injury in ALD. Here, we tested the hypothesis that treatment with the active microbial metabolite, UroA, could enhance gut barrier function and mitigate EtOH-induced gut barrier dysfunction, steatosis, and hepatitis. Our data suggest that UroA treatment protects against increased EtOH-induced barrier permeability by upregulating tight junction proteins in *in vitro* models. UroA treatment protected mice from alcohol-induced liver injury in both the acute and chronic models. Importantly, UroA treatment reduced EtOH-induced gut permeability and protected against disruption of tight junction proteins. Similarly, UroA treatment reduced the levels of hepatic inflammatory cytokines and triglycerides. The pathophysiology of ALD is linked to alcohol-induced gut barrier dysfunction, with disruption of TJPs, eventually leading to increased uptake of gut-derived toxins/endotoxins, such as LPS (Rao, 2009; Parlesak et al., 2000; Rao et al., 2004); however, the precise mechanism is poorly understood. *In vitro* studies have revealed that ethanol metabolites such as acetaldehyde can activate tyrosine kinase and phosphatase 2A by inhibiting tyrosine phosphatase (Elamin et al., 2012; Dunagan et al., 2012). Additionally, chronic alcohol consumption increases ROS levels by modulating intestinal CYP2E1 levels and disrupting the gut barrier, potentially by destabilizing tight junction proteins (Wang et al., 2020). Therefore, EtOH-induced ROS production might increase gut permeability. Importantly, AHR ligands have been reported to activate the *Ahr*-*Nrf2* gene battery, including UroA (Singh et al., 2019), thereby protecting the antioxidant pathway from ROS-induced damage.

AHR was initially discovered as a high-affinity receptor for the environmental toxicant 2,3,7,8-tetrachlorodibenzo-*p*-dioxin (TCDD) and was responsible for mediating adverse events, including multiple organ failure (Poland and Knutson, 1982; Patrizi and Siciliani de Cumis, 2018; Mohsenzadeh et al., 2018). However, recent studies have highlighted certain benefits of AHR activation by tryptophan metabolites in diverse mouse disease (Jin et al., 2014; Hubbard et al., 2015; Gao et al., 2018; Gargaro et al., 2021). Recent studies have supported the concept of selective AHR modulators (SAHRMs), in which AHR ligands exhibit variable tissue-, organ-, and species-specific genomic and functional AHR-dependent activities. SAHRMs are defined as being capable of altering transcriptional activities, at least in part, independently of dioxin response element (DRE) binding (Safe et al., 2018; Murray et al., 2010). We suggest that AHR activation by microbial metabolites facilitates bidirectional communication between the host and the microbiota, thereby leading to significant physiological changes through inter-organ axes in health and various disease conditions. Our previous studies demonstrated that UroA mediates its activity in an AHR-dependent manner and protects the gut barrier in preclinical colitis models (Singh et al., 2019). Recently, Hendrikx *et al.* showed that alcohol-fed mice had lower mean amounts of AHR ligands (i.e., tryptophan metabolites) such as indole acetic acid (IAA) or indole-3-sulfate in the small intestine (Hendrikx et al., 2019). Importantly, in a clinical setting, the levels of AHR ligands, such as IAA or indole-3-lactic acid, in stool samples were significantly reduced in patients with alcohol-associated hepatitis compared with controls (Gao et al., 2020). Therefore, we postulated that supplementation with beneficial AHR ligands would attenuate the development and progression of ALD. Our data from *Ahr* ^*−/−*^ mice demonstrated that UroA treatment protected mice from EtOH-induced increase in gut permeability in an AHR-dependent manner. UroA failed to reduce EtOH-induced ALT, TNF-α, and IL-6 levels in *Ahr* ^*−/−*^ mice. To delineate the cell-specific role of AHR in UroA-mediated protection against ALD, we tested its therapeutic activity in *Ahr* ^*ΔIEC*^ mice. Deletion of AHR in intestinal epithelial cells leads to increased damage in both the intestine and the liver upon EtOH exposure. Interestingly, UroA treatment failed to reduce EtOH-induced AST and ALT levels as well as inflammatory cytokines in *Ahr* ^*ΔIEC*^ mice. These results demonstrate that UroA has a significant effect on the gut, which could potentially translate into beneficial effects in ALD. Overall, the results suggest that UroA requires intestinal epithelial AHR to mediate gut barrier protective activities and that AHR in the liver is required for the progression of ALD.

We also observed that UroA treatment reduced overall triglyceride levels, even in *Ahr* ^*ΔIEC*^ mice, indicating that UroA may directly influence lipid metabolism. In addition, treatment with UroA significantly reduced EtOH-induced lipid accumulation in hepatocytes as well as hepatic triglycerides and cholesterol esters in mice fed with EtOH. In support of these observations, UroA treatment also downregulated the genes involved in lipid metabolism, *Srebp-1c* and *Ppar-γ,* in EtOH-fed mice. Furthermore, UroA reduced lipid accumulation in the hepatocyte cell culture system, indicating direct effects of UroA on lipid metabolism. Sirtuin 1 (SIRT1), an NAD^+^-dependent deacetylase, inhibits hepatic lipogenesis, stimulates fatty acid β-oxidation, and maintains cholesterol and bile acid levels (Ren et al., 2020). It has been shown that EtOH-mediated inhibition of SIRT1 or its deletion promotes the development of alcohol-associated fatty liver disease (Ren et al., 2020; You et al., 2015; Yin et al., 2014). Notably, treatment with UroA-induced sirtuin 1 (Sirt1)-AMP-activated kinase (AMPK) pathways in a variety of cells and models, including BV2 microglia (Velagapudi et al., 2019), adipocytes, hepatocytes (Kang et al., 2016), and skeletal muscle (Ghosh et al., 2020), attenuated D-galactose-induced brain aging in mice (Chen et al., 2019). UroA also attenuated hepatic TG accumulation in mice fed a high-fat diet (Tone et al., 2019). Based on these observations, we postulate that UroA treatment potentially downregulates EtOH-induced lipid accumulation in mouse livers by increasing sirtuin 1 (Sirt1)-AMP-activated protein kinase (AMPSIR1) pathways. SIRT1-AMPK mediates its activities by disrupting signaling pathways largely mediated by various transcriptional regulators and several co-regulators, including SREBP-1c and PPARα (Ren et al., 2020). Our data suggest that UroA treatment downregulates EtOH-induced expression of the transcription factors *Srebp-1c, Scd-1* and *Ppar-γ in vivo* models.

Our study has some limitations. No animal model has fully recapitulated the clinical and histological findings of alcohol-associated hepatitis. Therefore, we used several animal models of ALD as well as cell lines to validate the consistency of our therapeutic interventions. We only explored intestinal AHR knock-out mice and did not evaluate AHR deletion in other organs, such as the liver, which is a future direction. The estimation of liver cell death based on AHR activity will be a prominent future direction. This study also resembles the early-stage ALD staging conditions of human pathology. Finally, we did not validate our findings in humans, who are currently being evaluated.

In summary, these studies demonstrated that UroA mediates its activities in both intestines and regulates intestinal permeability and lipid metabolism in an AHR-dependent manner. The molecular mechanisms and signal transduction pathways responsible for these beneficial effects require further in-depth investigations in humans. Although our study provides compelling evidence that UroA can protect against alcohol-associated liver disease (ALD) by modulating the gut-liver axis, there are some limitations to consider. First, we acknowledge that our mechanistic study focused on intestinal epithelial AHR and that liver-specific AHR responses are still incomplete in terms of the impact of ethanol metabolism and UroA effect. Although the global deletion of *Ahr* and intestinal epithelial cell-specific *Ahr* deletion mouse (*Ahr* ^*ΔIEC*^) strains are informative, future experiments would benefit from using different ALD models in tissue-specific deleted mice to elucidate the precise tissue-specific contributions.

## Impact and implications

Recent studies highlight the importance of the microbial metabolite Urolithin A (UroA) in attenuating alcohol-induced gut barrier dysfunction, hepatitis, and lipogenesis. Here, we elucidated the mechanisms by which UroA protects against alcohol-induced intestinal and liver injury in an intestinal epithelial cell-aryl hydrocarbon receptor (AHR)-dependent manner. These findings highlight the potential of UroA for translational application in preventing alcohol-associated intestinal and liver diseases.

## Data Availability

The original contributions presented in the study are included in the article/Supplementary Material, further inquiries can be directed to the corresponding author.

## Supplementary material

SUPPLEMENTARY FIGURE S1 UroA performs better as a gut protective molecule compared to other EA metabolites. **(A)** Chemical structures of microbial metabolites of ellagic acid (EA). **(B,C)** Monolayered Caco2 cells on transwell membrane were pre-treated with vehicle, EA, UroA, UorB or UroC (50 μM) for 24 h followed by addition of EtOH (40 mM) for 24 h. FITC-dextran permeability assay **(B)** and the TEER **(C)** values were determined. **(D)** Representative additional Western blot for ZO-1 is shown from experiment of Figure 1E.

SUPPLEMENTARY FIGURE S2 Treatment with UroA reduced EtOH-induced junctional protein damage in the binge model. Expression of tight junction proteins OCLN was measured by Western blots experiment of Figure 2 and quantified using ImageJ software.

SUPPLEMENTARY FIGURE S3 UroA protects against acute alcohol-induced gut barrier dysfunction and liver damage. **(A)** 8-week old male C57BL/6J mice (n = 5) were treated orally with EtOH (5 g/kg) at 0, 12 and 24 h. Mice were treated with Veh or UroA at 4 and 26 h and euthanized at 36 h. FITC-dextran was orally administered 4 h prior to euthanization **(B)** Fecal albumin **(C)** Serum FITC-dextran **(D)** Serum ALT **(E)** Serum AST **(F)** Serum TNF-α **(G)** Serum IL-1β **(H)** Liver TNF-α **(I)** Liver IL-1β were measured using appropriate ELISA kits. **(J)** Expression of tight junction proteins CLDN4 were measured by Western blots in the small intestines and colon tissues.

SUPPLEMENTARY FIGURE S4 Characterization of cell-specific *Ahr*-deleted transgenic mice. *AhrVillin* ^*Cre*^: Top panel: Confocal images of colon sections of C57BL/6, *AhR* ^*fx*^ *Villin* ^*Cre*^ and *Ahr* ^*−/−*^ mice stained with AHR antibody, followed by secondary antibody tagged with Alexa 594. Nucleus stained with DAPI. *Bottom Panel*: AHR expression in different organs isolated from WT and *AhR* ^*fx*^ *Villin* ^*Cre*^ mice was determined by Western blots. Data show deletion of AHR in the intestines of *AhR* ^*fx*^ *Villin* ^*Cre*^ mice.

## Glossary

AALD: Alcohol-associated liver disease
AHR: Aryl hydrocarbon receptor
***Ahr*** ^***−/−***^: Aryl hydrocarbon receptor Knock out
***Ahr*** ^***fl/fl***^: Aryl hydrocarbon receptor floxed
***Ahr*** ^**△ IEC**^: Aryl hydrocarbon receptor deleted in intestinal epithelial cells
ALT: Alanine transaminase
AMP: Adenosine monophosphate
AMPK: AMP-activated kinase
AST: Aspartate aminotransferase
CEs: cholesteryl esters
CLDN1: Claudin-1
CLDN4: Claudin 4
CMC: Carboxy methyl cellulose.
CYP2E1: Cytochrome P450 2E1
DMEM: Dulbecco’s Modified Eagle’s Medium
DRE: Dioxin response element
EA: Ellagic acid
ELISA: enzyme-linked immunosorbent assay
EMEM: Eagle’s minimum essential medium
ET: Ellagitannin
EtOH: Ethyl alcohol
FITC: Fluorescein isothiocyanate
GI: Gastrointestinal
HBSS: Hanks' Balanced Salt Solution
IAA: Indole acetic acid
IECs: Intestinal epithelial cells
IFN-γ: Interferon gamma
IL-1β: Interleukin 1 beta
IL-6: Interleukin 6
LAL: Limulus Amebocyte Lysate assay
MLC: Myosin light chain
MLCK: Myosin light chain kinase
µM: Micromolar
NIAAA: National Institute on Alcohol Abuse and Alcoholism
NRF2: Nuclear factor erythroid 2-related factor 2
OCLN: Occludin
PBS: Phosphate Buffered Saline
PF: Pair fed
PPARγ: Peroxisome proliferator-activated receptor gamma
ROS: Reactive oxygen species.
RT-PCR: Real time polymerase chain reaction.
SAHRMs: Selective AHR modulators
SIRT1: Sirtuin 1
SREBP-1c: Sterol regulatory element-binding protein -1c
TCDD: 2,3,7,8-tetrachlorodibenzo-*p*-dioxin
TEER: transepithelial electrical resistance
TG: Triglycerides
TJP: Tight junction proteins
TNF-α: Tumor necrosis factor alpha
UroA: Urolithin A
ZO-1: zonula occludens
