# Supplementary figures and images for: Urolithin A regulates gut: liver axis to ameliorate alcohol-associated liver disease
Source: Front Pharmacol. 2026 Jan 19;16:1706111. doi: 10.3389/fphar.2025.1706111 (PMC12862256; doi:10.3389/fphar.2025.1706111)

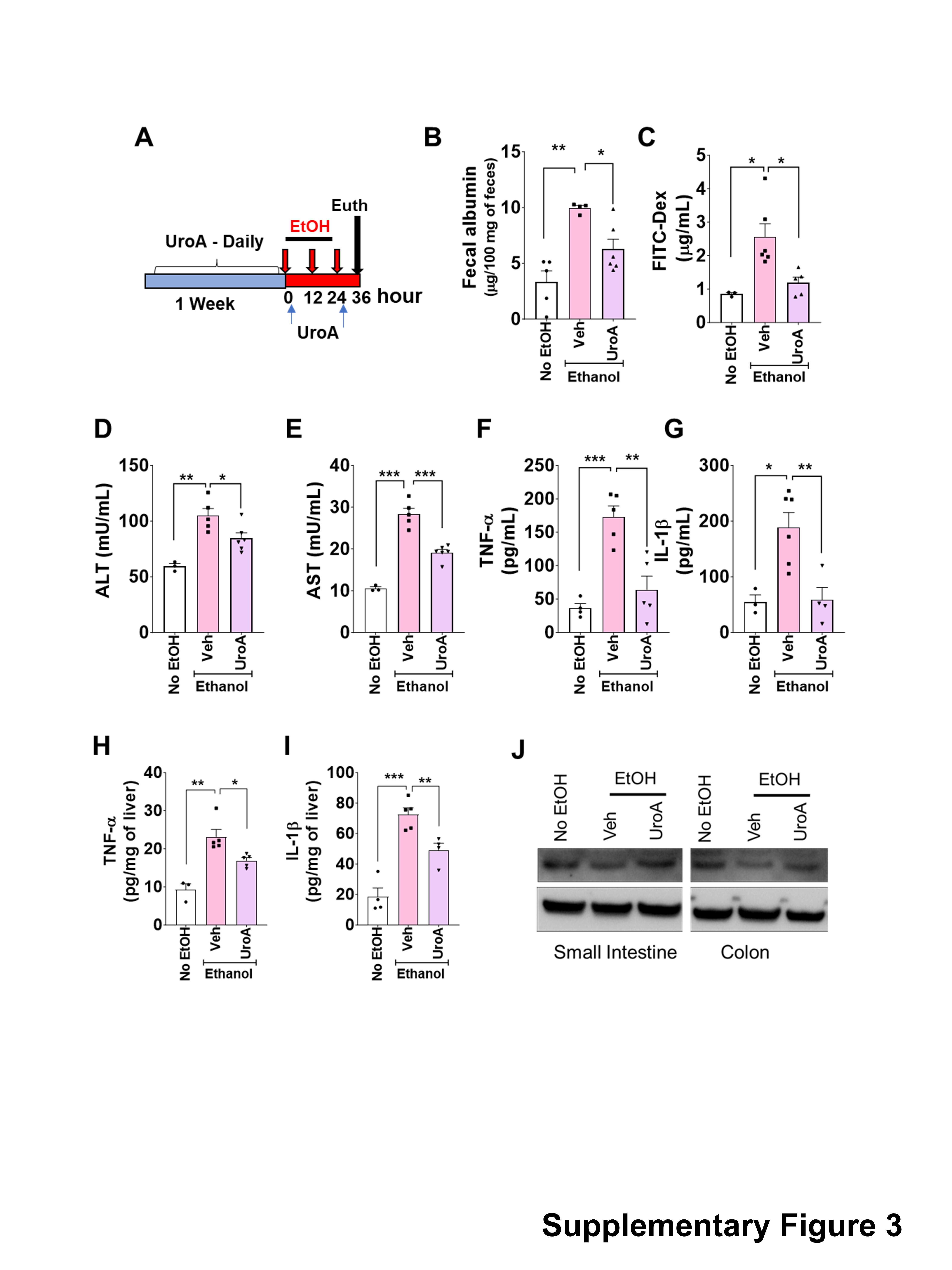

Supplement: Supplementary file 1 [file Image3.jpeg]

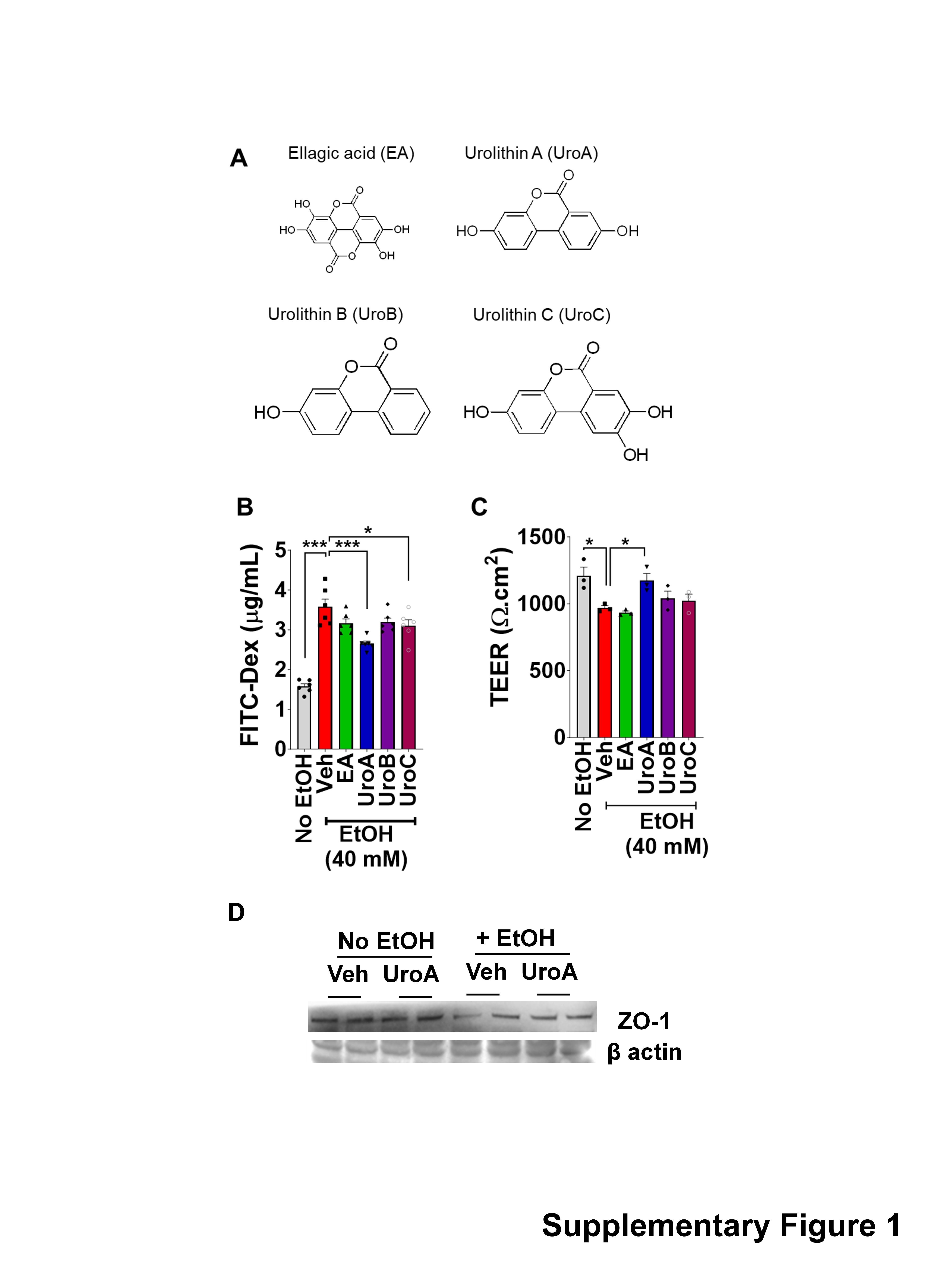

Supplement: Supplementary file 2 [file Image1.jpeg]

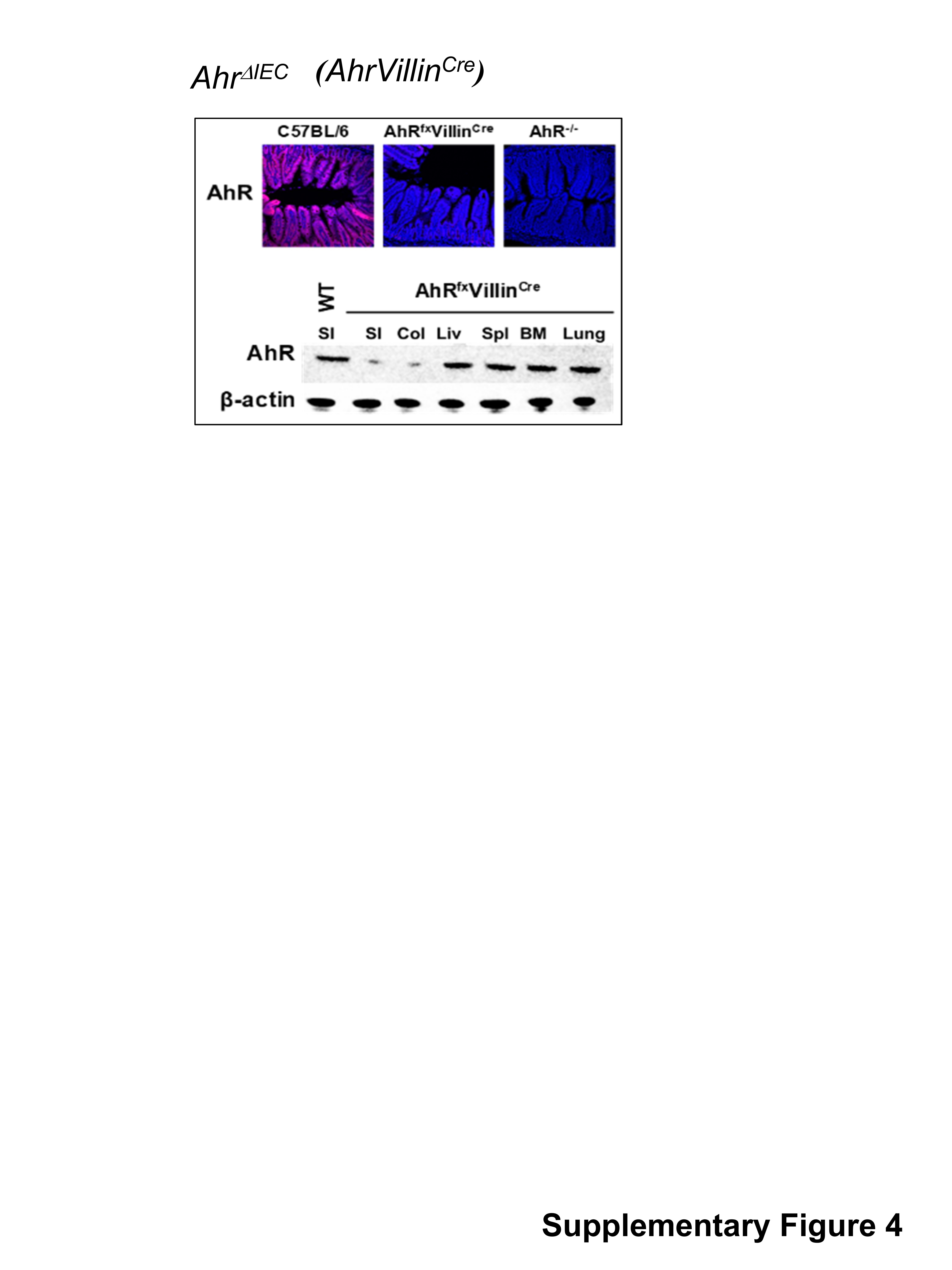

Supplement: Supplementary file 3 [file Image4.jpeg]

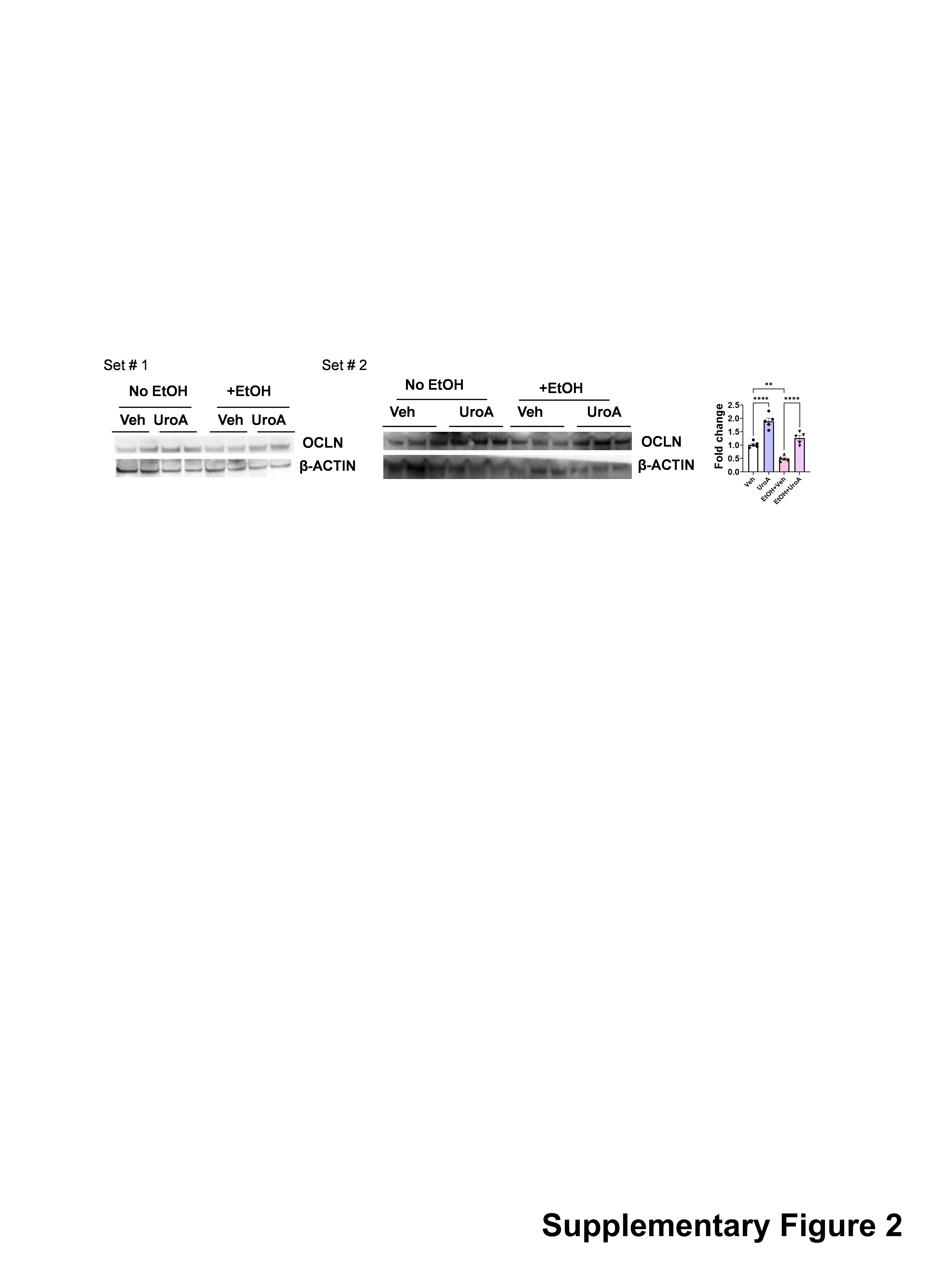

Supplement: Supplementary file 4 [file Image2.jpeg]
